# Cancer immunotherapy; integration of T cell biology with nanogel- and vector-technology in translational research

**DOI:** 10.1186/ar3564

**Published:** 2012-02-09

**Authors:** Hiroshi Shiku

**Affiliations:** 1 Dept. of Cancer Vaccine and Immuno-Gene Therapy, Mie University Graduate School of Medicine, Japan

## 

Molecular definition of cancer specific antigens recognized by T cells opened an approach to develop cancer specific immunotherapy. Through a series of key findings in cancer immunology, for development of effective therapy major effort has been directed to how to induce T cells with fine specificity, sufficient quantity and high quality in hosts.

We intended to integrate immunobiological strategy of T cells with two technologies, nanogel technology and retroviral vector technology for translational research of cancer immunotherapy. Cholesterol-bearing hydrophobizedpullulan (CHP), physically cross-linked nanogels by self-assembly, form nanoparticle complex with protein in water.We found that antigen protein with multiple T cell epitopes, when complexed with CHP, was efficiently transported to lymph nodes and well captured by antigen presenting cells such as dendritic cells and macrophages leading to cross presentation. Hence, CHP-antigen protein complex may become excellent cancer vaccine to induce both CD8^+ ^killer T cells and CD4^+ ^helper T cells of high quality.

Intrinsic weakness of insufficiency in number of cancer specific T cells in hosts, prompted us to develop adoptive T cell therapy with lymphocytes engineered to possess cancer specificity. For this purpose, we developed novel retroviral vectors to highly express exogenously transduced cancer specific T cell receptor (TCR), yet suppressing expression of endogenous polyclonal TCR. This approach allowed us to prepare T cells with finer specificity of expressed TCR. In addition, use of RetroNectin^®^, a recombinant fragment of fibronectin opened a way to ex vivo prepare T cells of sufficient quantity and good quality for clinical use.

Translational clinical trials of these cancer vaccine and adoptive T cell therapy are now on-going.

An open innovation to promote fusion of different fields of science and technology played an essential role in our development of cancer immunotherapy.

